# Clinical Efficacy of Atomoxetine Hydrochloride Combined with Electroencephalogram Biofeedback in Attention-Deficit/Hyperactivity Disorder in Children

**DOI:** 10.1523/ENEURO.0371-24.2025

**Published:** 2025-04-01

**Authors:** Xinyue Liu, Xiaoliang Li, Limin Liu, Xiao Sun, Zhe Yu

**Affiliations:** Department of Paediatrics, Xingtai Central Hospital, Xingtai, Hebei 054000, China

**Keywords:** atomoxetine hydrochloride, attention, attention-deficit/hyperactivity disorder, children, clinical efficacy, control ability, electroencephalogram biofeedback

## Abstract

Attention-deficit/hyperactivity disorder (ADHD) adversely affects the learning, social interaction, and daily living of affected children. Atomoxetine (ATX) hydrochloride (HCI) has been widely used in clinical practice. Electroencephalogram (EEG) biofeedback, as a nonpharmacological treatment approach, has also demonstrated potential in improving symptoms in children with ADHD. We aimed to investigate the clinical efficacy of combining ATX HCI with EEG biofeedback in the treatment of ADHD in children. We hypothesized that this combined therapy would be more effective in alleviating symptoms in children with ADHD. Ninety children with ADHD were randomly separated into the control group (receiving ATX HCI treatment for 12 weeks) and study group (receiving ATX HCI treatment for 12 weeks combined with 60 sessions of EEG biofeedback treatment; *n* = 45). Swanson, Nolan, and Pelham-IV (SNAP-IV) rating scale scores, integrated visual and auditory continuous performance test results, Conners parent symptom questionnaire (PSQ) scores, and adverse reactions were counted. After 12 weeks of treatment, SNAP-IV scores were lower in both groups and were much lower in the study group; full-scale attention quotient and full-scale response control quotient scores were elevated in both groups and were much higher in the study group; PSQ scores were lower in both groups and were much lower in the study group (all *p* < 0.05). During the treatment period, there was no difference in the incidence of adverse reactions between both groups (*p* > 0.05). The treatment combination of ATX HCI and EEG biofeedback is effective for children with ADHD, improving their behavioral issues and psychological conditions.

## Significance Statement

This study lays a foundation to explore the combined effects of atomoxetine hydrochloride and electroencephalogram biofeedback in children with ADHD.

## Introduction

Attention-deficit/hyperactivity disorder (ADHD) is the most common neurobehavioral disease in children. This disorder is mainly manifested by developmentally inappropriate inattention and hyperactivity/impulsivity, causing impairment in one or more aspects of academic, emotional, and social function (Rajaprakash and Leppert, 2022). As a biology-based neurodevelopmental alteration, ADHD starts in childhood and may persist during adolescence-youth (Teran Prieto, 2020). Management primarily confines itself to behavioral and pharmacological interventions (Rajaprakash and Leppert, 2022).

Atomoxetine (ATX) represents the inaugural nonstimulant medication designed to treat ADHD in both children and adults (Fu et al., 2023). The data presented by Suravi Patra et al. indicate that ATX can alleviate symptoms of ADHD, including hyperactivity and inattention (Patra et al., 2019). Moreover, ATX has proven to be effective in alleviating anxiety symptoms among children and adolescents diagnosed with ADHD (Khoodoruth et al., 2022). Meanwhile, ATX is also demonstrated to have an association with improved patient-reported clinical responses and quality of life in patients with ADHD (Elliott et al., 2020). However, at the same time, ATX is also accompanied by a series of side effects, such as nausea, vomiting, decreased sleep, and loss of appetite (Patra et al., 2019). These adverse effects have somewhat restricted the broad application of ATX and driven the medical field to explore safer and more efficacious therapeutic approaches.

Electroencephalogram (EEG) biofeedback, also known as neurofeedback, serves as a treatment option for a range of neurological, somatic, and mental conditions. In the realm of psychiatry, it has been clinically applied to address disorders such as depression, ADHD, schizophrenia, substance abuse, neuroses, and Alzheimer's disease. Studies suggest that the neuromodulatory impact of this therapy exerts a beneficial influence on cognitive functions, mood regulation, and anxiety levels (Markiewcz, 2017). As a nonpharmacological treatment approach, EEG biofeedback has gradually emerged as a prominent option in the treatment of ADHD in recent years. Biofeedback involves training individuals to control their physiological responses. Its various forms encompass electromyography, electrodermal activity, skin temperature regulation, heart rate and heart rate variability management, EEG feedback, and blood oxygenation level-dependent signals obtained via functional magnetic resonance imaging (Tolin et al., 2020). Neurofeedback represents a type of biofeedback where real-time information about brain activity is provided to an individual as feedback (Dousset et al., 2020), which allows the user to take control of their neuronal activity and, consequently, fosters long-term brain plasticity. It presents itself as an intriguing tool for addressing brain disorders (Marchi et al., 2024). Dazhi Cheng and his colleagues have confirmed that EEG biofeedback has the potential to enhance key cognitive abilities in children with idiopathic epilepsy syndromes who also have ADHD (Cheng et al., 2024). Furthermore, the report states that the use of pestle needle therapy combined with EEG biofeedback and methylphenidate in children with ADHD can improve EEG wave patterns and sleep quality, as well as regulate serum indicators (Wang et al., 2024). Despite demonstrating certain efficacy in the treatment of ADHD, clinical studies on the combined therapy of ATX and EEG biofeedback remain relatively scarce. It is noteworthy that medications may enhance the ability of children with ADHD to learn from EEG biofeedback. In other words, there may be a synergistic effect between pharmacological and nonpharmacological treatments (Lin et al., 2022). ATX improves ADHD symptoms by modulating neurotransmitter levels, while EEG biofeedback achieves a similar effect by regulating brain electrical activity. The two possess a certain degree of complementarity in their mechanisms of action, suggesting that combined therapy may yield better therapeutic outcomes.

The EEG biofeedback treatment in this study consisted of six phases, including baseline testing, two rounds of theta and beta wave training, parameter readjustment, and baseline retesting. Each training session lasts ∼30 min, with five sessions per week for a total of 12 weeks of treatment, amounting to 60 sessions in total. This study aimed to determine the clinical efficacy of ATX hydrochloride (HCI) and EEG biofeedback in children with ADHD.

## Materials and Methods

### Ethics statement

The study was approved by the ethics committee of our hospital. The children’s families signed the written informed consent form.

### Participants

Ninety children with ADHD admitted to our hospital from January 2020 to December 2022 were included in this randomized double-blinded controlled trial.

The inclusion criteria were as follows: (1) Children who met the diagnostic criteria for ADHD in the Diagnostic and Statistical Manual of Mental Disorders, Fifth Edition (First, 2013); (2) those aged 6–12 years old and with normal intelligence as measured by the Wechsler Intelligence Scale; (3) those without physical and neurological diseases; (4) those received no other treatment within the past 2 weeks and taken no psychiatric, central nervous system drugs; (5) those without skin lesions, ulcers, or infections in the treatment area; and (6) those able to take the medication according to the doctor’s instructions and whose parents can supervise the medication and treatment.

The exclusion criteria were as follows: (1) children who have dropped out of compulsory education; (2) those whose imaging showed organic brain lesions; (3) those combined with psychoneuropathy, mental retardation, affective disorders, and audio–visual disorders; (4) those combined with phenylketonuria, pediatric chorea, and fragile X syndrome; (5) those combined with severe impairment or insufficiency in cardiopulmonary, hepatic, renal, and hematopoietic system; (6) those with self-harm and suicidal tendencies; and (7) those with contraindications to the medications and EEG biofeedback therapy used in this study.

During the course of this study, there were no withdrawal or dropout cases in either group.

After enrollment, participants were randomly allocated to either the study group or control group. Children in the control group were treated with ATX HCI, and those in the study group were treated with EEG biofeedback on top of the control group. In the control group, the children were aged 6–12 years, with 38 males and 7 females. In the study group, the age range was also 6–12 years, with 35 males and 10 females. The baseline information of the two groups was not statistically different (*p* > 0.05; Table 1) and was comparable.

**Table 1.** Baseline information between the two groups

Indicators	The control group (*n* = 45)	The study group (*n* = 45)	*p* value
Age of children (years)	7.00 (7.00, 9.00)	8.00 (7.00, 9.00)	0.754
Gender of children			0.419
Male	38 (84.44)	35 (77.78)	
Female	7 (15.56)	10 (22.22)	
Disease duration of children			0.479
Within 1 year	12 (26.67)	8 (17.78)	
Within 2 years	17 (37.78)	22 (48.89)	
Within 3 years	13 (28.89)	14 (31.11)	
Within 4 years	3 (6.67)	1 (2.22)	
Age of guardian (years)	34.09 ± 2.56	34.89 ± 3.02	0.178
Gender of guardian			0.245
Male	9 (20.00)	5 (11.11)	
Female	36 (80.00)	40 (88.89)	
Educational level of guardian			0.685
Primary school	14 (31.11)	16 (35.56)	
Middle school	19 (42.22)	15 (33.33)	
University	12 (26.67)	14 (31.11)	

### Procedure and intervention

The control group received ATX HCI treatment, while the study group received ATX HCI combined with EEG biofeedback treatment.

ATX HCI treatment: The patients were treated with oral ATX HCI (manufactured by Henan Topfond Pharmaceutical, H20120098). The initial dosage was 0.5  mg/kg per day, administered once daily after meals. Depending on the individual's condition, the dosage was gradually increased to 1.2  mg/kg after 3 d and maintained for 12 weeks.

EEG biofeedback treatment: The EEG biofeedback instrument was manufactured by Nanjing Weisi Medical Technology. The EEG biofeedback treatment process consisted of six stages: Stage 1, baseline testing. This stage primarily involved monitoring the initial EEG threshold values to adjust parameters during subsequent treatments. It lasted for ∼2 min. Stage 2, theta wave training. Based on changes in EEG patterns, adjustments were made to the training method. This stage lasted for 5 min. Stage 3, beta wave training. Similar to Stage 2, adjustments were made according to EEG pattern changes. This stage also lasted for 5 min. Stage 4, continued theta wave training. This stage further reinforced theta wave training. Stage 5, continued beta wave training. This stage continued beta wave training. Stage 6, recalibration of parameters and baseline testing. A baseline test was conducted again to compare the results with the first measurement. After each training session, the Inﬁniti300C feedback instrument will provide individual results for each training. Through the interface of the user data processing center, users can view their performance records and improvements in attention, as well as analyze and compare data before and after the treatment. Throughout the treatment process, the therapist monitored the child on a one-to-one basis. Each training session lasts ∼30 min, with five sessions per week for a total of 12 weeks of treatment, amounting to 60 sessions in total.

All pediatric patients undergo medical checkups every 4 weeks.

### Observation indicators

1. Swanson, Nolan, and Pelham-IV (SNAP-IV) rating scales: The SNAP-IV scores of the two groups before treatment and after 12 weeks of treatment were compared. There were 26 items including three factors of attention, hyperactivity/impulsivity, and oppositional defiance. Each item was scored by a four-level scoring system ranging from zero to three points. The score of each factor was equal to the total score of each factor divided by the number of items. Decreased scores indicated alleviated symptoms of ADHD.
2. Integrated visual and auditory continuous performance test (IVA-CPT): IVA-CPT results were compared between the two groups before treatment and after 12 weeks of treatment. This contained two test systems of visual and auditory stimulation, which lasted for 12–15 min, and the program automatically recorded the reaction time, the number of errors, and the number of omissions. After the test was completed, the system calculated the full-scale attention quotient (FAQ) and the full-scale response control quotient (FRCQ).
3. Conners parent symptom questionnaire (PSQ): PSQ scores were compared between the two groups before treatment and after 12 weeks of treatment. The PSQ contained a total of 48 entries for six factors, including hyperactivity index, impulsive hyperactivity, physical and mental disorders, learning problems, behavioral problems and anxiety, and each entry adopted a four-level scoring system from zero to three points. The score of each factor was equal to the total score of each factor divided by the number of entries, and the lower the score was, the better the children’s behavioral performance and psychological status.
4. Adverse reactions: at the 2nd week of treatment and the 12th week of treatment, the occurrence of adverse reactions in the two groups of children was monitored.

### Statistics

Statistical analysis was performed by implementing the SPSS 26.0 software. Qualitative data were depicted by [*n* (%)], and the *χ*^2^ test was performed. The Kolmogorov–Smirnov test was used to check the normality of quantitative data. Quantitative data that obeyed a normal distribution were described using $\bar{x}$ ± s, and the *t* test was performed. Quantitative data that did not satisfy a normal distribution were expressed as median [M (P25, P75)], the Wilcoxon rank-sum test was adopted for comparisons within groups before and after treatment, and the Mann–Whitney *U* test was employed for comparisons between groups. *p* < 0.05 was considered a statistically significant difference.

## Results

### SNAP-IV scores

Compared with pretreatment, the SNAP-IV scores for each subscale (attention, hyperactivity/impulsivity, and oppositional defiance) in both the control group and the study group decreased after treatment (*p* < 0.05). When comparing the two groups after treatment, the scores in the study group were much lower, showing a significant difference (*p* < 0.05; Table 2). This indicates that both treatment regimens in the control group and the study group can reduce SNAP-IV scores, but the latter is more effective than the former.

**Table 2.** SNAP-IV scores between the two groups (points)

Indicators	Time	The control group (*n* = 45)	The study group (*n* = 45)	*p* value
Attention	Before treatment	2.34 ± 0.34	2.30 ± 0.41	0.625
	After treatment	1.84 ± 0.30^a^	1.64 ± 0.27^a^	0.001
Hyperactivity/impulsivity	Before treatment	1.89 (1.62, 2.11)	1.89 (1.67, 2.11)	0.987
	After treatment	1.44 (1.11, 1.67)^a^	1.33 (1.00, 1.44)^a^	0.026
Oppositional defiance	Before treatment	1.50 (1.32, 1.88)	1.63 (1.25, 1.88)	0.974
	After treatment	1.25 (1.13, 1.50)^a^	1.13 (0.88, 1.38)^a^	0.032

a *p* < 0.05 versus the same group before treatment.

### IVA-CPT results

Compared with pretreatment, the FAQ and FRCQ scores of both the control group and the study group increased after treatment (*p* < 0.05). When comparing the two groups after treatment, the FAQ and FRCQ scores were much higher in the study group, indicating a significant difference (*p* < 0.05; Table 3). This suggests that both the control group and the study group can improve the IVA-CPT test scores of children with ADHD, with the study group showing a better effect than the control group.

**Table 3.** IVA-CPT results between the two groups (points)

Indicators	Time	The control group (*n* = 45)	The study group (*n* = 45)	*p* value
FAQ	Before treatment	72.00 (67.00, 75.00)	71.00 (67.00, 73.50)	0.365
	After treatment	85.00 (82.00, 88.00)^a^	90.00 (86.00, 92.50)^a^	<0.001
FRCQ	Before treatment	78.00 (74.00, 80.00)	78.00 (74.00, 80.00)	0.824
	After treatment	95.00 (90.00, 97.50)^a^	99.00 (94.00, 101.50)^a^	0.001

a *p* < 0.05 versus the same group before treatment.

### PSQ scores

Compared with pretreatment, the PSQ scores for each category (including hyperactivity index, impulsive hyperactivity, physical and mental disorders, learning problems, behavioral problems, and anxiety scores) in both the control group and the study group decreased after treatment (*p* < 0.05). When comparing the two groups after treatment, the study group showed more pronounced decreases in hyperactivity index, impulsive hyperactivity, physical and mental disorders, learning problems, behavioral problems, and anxiety scores, with significant differences between groups (*p* < 0.05; Table 4). This indicates that both the control group and the study group can reduce PSQ scores in children with ADHD, with the study group showing a better effect than the control group.

**Table 4.** PSQ scores between the two groups (points)

Indicators	Time	The control group (*n* = 45)	The study group (*n* = 45)	*p* value
Hyperactivity index	Before treatment	1.70 (1.40, 1.90)	1.70 (1.40, 1.80)	0.658
	After treatment	1.30 (1.05, 1.50)^a^	1.00 (0.80, 1.20)^a^	<0.001
Impulsive hyperactivity	Before treatment	1.75 (1.25, 1.75)	1.50 (1.25, 1.75)	0.536
	After treatment	1.00 (1.00, 1.25)^a^	1.00 (0.75, 1.00)^a^	0.001
Physical and mental disorders	Before treatment	0.40 (0.40, 0.60)	0.60 (0.40, 0.60)	0.785
	After treatment	0.20 (0.20, 0.40)^a^	0.20 (0.20, 0.20)^a^	0.001
Learning problems	Before treatment	2.00 (1.63, 2.25)	2.00 (1.50, 2.13)	0.666
	After treatment	1.25 (1.00, 1.50)^a^	1.00 (0.75, 1.13)^a^	<0.001
Behavioral problems	Before treatment	1.08 (0.88, 1.21)	1.08 (0.79, 1.17)	0.472
	After treatment	0.83 (0.54, 1.00)^a^	0.58 (0.42, 0.67)^a^	<0.001
Anxiety	Before treatment	0.50 (0.25, 0.50)	0.50 (0.25, 0.50)	0.734
	After treatment	0.25 (0.25, 0.25)^a^	0.25 (0.00, 0.25)^a^	<0.001

a *p* < 0.05 versus the same group before treatment.

### Adverse reaction rate

As a nonpharmacological therapy, EEG biofeedback has no side effects. Therefore, the adverse reactions observed in both groups of children were due to the administration of ATX HCI. Some children in both the control group and the study group experienced adverse reactions such as decreased appetite, drowsiness, and headache within 2 weeks of taking ATX HCI. However, these adverse reactions were effectively alleviated after reducing the dosage or changing the administration time to after dinner. The differences in adverse reactions between the control group and the study group were not significant at the 2nd and 12th weeks of treatment (*p* > 0.05; Table 5).

**Table 5.** Adverse reaction rate between the two groups [*n* (%)]

Adverse reaction	Time	The control group (*n* = 45)	The study group (*n* = 45)	*p* value
Loss of appetite	The 2nd week	21 (46.67)	23 (51.11)	0.673
	The 12th week	8 (17.78)^a^	6 (13.33)^a^	0.561
Drowsiness	The 2nd week	20 (44.44)	18 (40.00)	0.670
	The 12th week	2 (4.44)^a^	1 (2.22)^a^	0.557
Headache	The 2nd week	7 (15.56)	10 (22.22)	0.419
	The 12th week	0 (0.00)^a^	0 (0.00)^a^	-
Nausea and vomiting	The 2nd week	3 (6.67)	2 (4.44)	0.645
	The 12th week	0 (0.00)	0 (0.00)	-
Emotional instability	The 2nd week	3 (6.67)	3 (6.67)	>0.999
	The 12th week	0 (0.00)	1 (2.22)	0.315
Constipation	The 2nd week	2 (4.44)	1 (2.22)	0.557
	The 12th week	0 (0.00)	0 (0.00)	-

a *p* < 0.05 versus the same group at the second week of treatment.

## Discussion

This study focused on the clinical effects of ATX HCI and EEG biofeedback in children with ADHD. Our study demonstrated that the treatment combination of ATX HCI and EEG biofeedback was effective for children with ADHD, significantly improving their behavioral issues and psychological conditions.

The research results indicated that after 12 weeks of treatment, both the control group receiving only ATX HCI treatment and the study group receiving ATX HCI combined with 60 sessions of EEG biofeedback treatment showed decreased SNAP-IV scores (attention, hyperactivity/impulsivity, and oppositional defiance). However, the SNAP-IV scores in the study group were lower than those in the control group. This suggests that both treatment regimens can reduce SNAP-IV scores, but compared with ATX HCI monotherapy, the combination of ATX HCI and EEG biofeedback significantly alleviates ADHD symptoms in children. As previously reported, EEG-based neurofeedback combined with medications has more benefits in the treatment of inattention symptoms in patients with ADHD versus medication alone (Lin et al., 2022). It is also demonstrated to improve inattention and hyperactivity/impulsivity scores in patients with ADHD (Micoulaud-Franchi et al., 2014). According to data, administering a combination of pestle needle therapy, EEG biofeedback, and methylphenidate to children with ADHD can lead to an enhancement in their IVA-CPT scores (Wang et al., 2024). These are similar to the findings of this study. Our results showed that both the FAQ and FRCQ scores of the control group and the study group increased after 12 weeks of treatment, with higher scores in the study group. This indicates that both ATX HCI treatment and the combination of ATX HCI and EEG biofeedback can improve IVA-CPT test scores, but the combination therapy is superior to ATX HCI monotherapy. In other words, the combination of ATX HCI and EEG biofeedback improves attention and control abilities in children with ADHD. A study demonstrates that starting from the fourth week of treatment, ATX is more effective than methylphenidate in alleviating anxiety symptoms (Snircova et al., 2016). After 12 weeks of treatment in this study, PSQ scores (including hyperactivity index, impulsive hyperactivity, psychosomatic disorders, learning problems, behavioral problems, and anxiety scores) decreased in both groups, with greater decreases in the study group across all PSQ categories. This suggests that both treatment regimens effectively reduce PSQ scores in children with ADHD, with the combination of ATX HCI and EEG biofeedback showing superior effects. This means that the combination therapy of ATX HCI and EEG biofeedback can enhance behavioral abilities and improve the psychological status of children with ADHD.

Another study has demonstrated that ATX is well tolerated, and adverse events are generally mild and transient; patients with ADHD often present abdominal pain, decreased appetite, as well as nausea and vomiting. ATX’s good safety and efficacy make it an effective treatment for ADHD and related comorbidities (Dell'Agnello et al., 2009). It is noteworthy that EEG biofeedback, as a nonpharmacological therapy, is noninvasive, safe, and free from side effects (Gong et al., 2021; Rice et al., 2024). Our study confirmed that some children in both the control group and the study group experienced adverse reactions within 2 weeks of taking ATX HCI. However, these adverse reactions were effectively alleviated after reducing the dosage or changing the administration time to after dinner. Furthermore, there was no significant difference in adverse reactions between the two groups at the 2nd and 12th weeks of treatment, indicating that adverse reactions did not worsen over time and that patients were able to gradually adapt to the treatment. This means that ATX HCI and EEG biofeedback are safe for treating ADHD in children. Data from Allan I. Levey et al. reveal that ATX treatment is safe and well tolerated and can achieve the goal of treating prodromal Alzheimer's disease (Levey et al., 2022). Maurizio Coppola et al.'s experiments confirm that ATX is both effective and safe for patients with co-occurring ADHD and alcohol dependence (Coppola and Mondola, 2018).

Hsien-Jane Chiu and her colleagues state that the effectiveness of surface EEG neurofeedback in improving sustained attention is satisfactory, particularly when beta wave enhancement is incorporated (Chiu et al., 2022). Based on the above results of this study, EEG biofeedback training enables patients to understand their brain state in real time and learn self-regulation through feedback signals. While ATX can alleviate ADHD symptoms, EEG biofeedback provides a long-term, self-driven regulatory mechanism that helps patients better manage their symptoms in daily life. Furthermore, when ATX is used in combination with EEG biofeedback, patients may be more willing to adhere to medication therapy due to their positive response to EEG biofeedback, thereby enhancing the overall treatment effect. Importantly, when ATX is combined with EEG biofeedback, doctors can adjust the drug dosage based on the patient's specific conditions to reduce the risk of side effects. In other words, EEG biofeedback complements ATX at the behavioral level, jointly enhancing the effectiveness of ADHD treatment. This combined therapy not only optimizes brain function but also improves patients’ self-regulation ability and adherence while reducing the occurrence of drug side effects. In terms of advantages, the combination of ATX HCI and EEG biofeedback treatment is effective and relatively safe. However, on the other hand, ATX HCI, as a prescription medication, may have higher costs. Additionally, EEG biofeedback treatment requires specialized equipment and trained personnel, increasing treatment costs. Moreover, it takes some time to observe significant efficacy with combination therapy, which can be a challenge for children with ADHD who need rapid symptom improvement.

### Conclusion and limitation

In summary, this research demonstrates that ATX HCI and EEG biofeedback in combination can treat ADHD in children, which can improve children’s attention and control ability, and in turn promote positive behavioral performance and psychological status. This study lays a foundation to explore the combined effects of ATX HCI and EEG biofeedback in children with ADHD. However, this study did not consider including control conditions such as a placebo group and a nonfeedback attention training group. This design limitation may introduce potential bias.

### Future prospects and suggestions

Future studies can consider incorporating these control conditions and expanding the sample size to further explore the specific mechanisms of the combination of ATX HCI and EEG biofeedback in treating ADHD, providing a theoretical basis for optimizing treatment regimens. At the same time, a more comprehensive assessment of the clinical efficacy of ATX HCI and EEG biofeedback in children with ADHD can be conducted to further enhance the scientificity and reliability of the research. Therefore, we offer some suggestions for future research. Strengthening the study of the mechanisms of combined therapy to uncover the biological basis of its efficacy is crucial. Although this study demonstrated the effectiveness of combined therapy, our understanding of its specific mechanisms of action remains limited. Future research can utilize neuroimaging, neurophysiology, and other techniques to deeply explore how ATX HCI and EEG biofeedback synergistically act on the brains of ADHD children, thereby improving their behavioral and psychological states. Furthermore, conducting large-scale, multicenter clinical trials is necessary to validate the efficacy and safety of combined therapy. Optimizing treatment regimens, exploring other effective treatments, and developing more comprehensive ADHD treatment plans that cater to the needs of different children are also important. Meanwhile, combined therapy may involve higher treatment costs, including medication costs and EEG biofeedback equipment costs. Future research can conduct cost–benefit analyses to assess the economy and feasibility of combined therapy compared with other treatment methods. Through these efforts, we hope to provide more effective, safer, and more personalized treatment options for ADHD children, improving their quality of life and driving continuous progress in research in this field.

## Synthesis

Reviewing Editor: Frederike Beyer, Queen Mary University of London Decisions are customarily a result of the Reviewing Editor and the peer reviewers coming together and discussing their recommendations until a consensus is reached. When revisions are invited, a fact-based synthesis statement explaining their decision and outlining what is needed to prepare a revision will be listed below. The following reviewer(s) agreed to reveal their identity: Luisa Himmelmeier.

On reviewing your revised manuscript, it struck me that the duration of the experiment has changed from 8 weeks in your original submission to 12 weeks in the revised version. please explain how this was done, did you decide to collect additional data based on reviewer comments? Was the study incomplete at the time of the original submission? This change in methodology appears to have changed your results and conclusions, which should be clearly explained and justified in your cover letter / rebuttal. If you are unsure about this point, I would encourage you to get in touch to check if a resubmission would likely be accepted for publication.

Besides this crucial aspect, if deciding to re-submit your manuscript to eNeuro, please address the following review comments:

Reviewer comments:

p. 2: The abstract should include a brief theoretical derivation that leads to the research question of the study. In addition, the duration of the ATX HCI treatment and the number of neurofeedback sessions in the combined experimental group should be stated more clearly. The hypotheses should be clearly formulated in the abstract: Were the authors concerned with a difference hypothesis, that the combined treatment of ATX HCI and NFB was more effective than ATX HC alone?

Inclusive wording: not ADHD children, but children with ADHD

p.2 l. 17: Does 'much higher in the study group' mean statistical significance?

p.2 ll.20: Do the adverse reactions refer to the ATX HC or also to the NFB?

p. 2 ll.22: choose more cautious wording, as there are enough studies that prove the inferiority of NFB compared to medication

p.3 l.36: more frequently observed in boys than in girls is inaccurate, there are gender differences with regard to compensation for symptoms, but girls with ADHD are often not diagnosed.

p.4: further differentiation should be made here with regard to the stated effectiveness of NFB in children with ADHD: what kind of NFB training was carried out over how many sessions and how was the effectiveness measured? At the end of the introduction, the setup of the NFP training should be clearly derived. In addition, the aspect that medication in some studies has increased the ability of children with ADHD to learn from NFB could be highlighted.

p.4 l. 80: Was the medication uniform in both groups or individually adapted to the height and weight of each child?

p.4 ll.81: here it would be good to indicate M and SD of the two groups and also to list the ADHD diagnoses (subtypes) with their distributions in the study sample.

p. 5 ll. 86: Who checked that the diagnostic criteria according to DSM-V were met? Test directors or physicians, psychologists or therapists independent of the study?

p.5 l.93: How was the 'good supervisory role' assessed and by whom?

p. 5 l.102: If there were no withdrawals and drop-outs, why are the criteria mentioned here in such detail? Wouldn't the description that there were none suffice?

p. 6, l. 111: The medication was administered by the child's parents? Every day at the same time? This is unclear and should be added. At what intervals were there medical check-ups?

p. 6, l.115 f.: What were the set reward frequencies of beta and theta as well as SMR? The combination of theta and beta as well as SMR does not correspond to any of the three standard protocols, which in turn requires a more precise description from my point of view. How were all the trainings included in the neurofeedback? Does this contradict the neurofeedback process from the introduction? Were there also standardized instructions and feedback animations? Couldn't the relaxation training before the session distort the effects? What were the preset numbers of the training? How many sessions of neurofeedback did the children receive?

p. 6, l.126: the sources for the test procedures are missing

p.7, l. 145: How was the care provided? Were adverse effects recorded by the test managers or were there fixed contacts with doctors?

p. 7, l.150: Were there any missing values in the questionnaires? How were these replaced? Were there any test cancellations in the CPT?

p. 7, l. 151: M should be defined more precisely. It should also be explained that the Mann-Whitney U test was used due to the violation of the requirements for parametric procedures and why exactly this test was chosen.

p.7, l.156: The breakdown by test procedure blurs the clarity of the presentation. I recommend dividing the results by test group. It can then be stated which results there were in the pre-post comparison of the respective study group across the respective results before the comparison between the study groups is drawn. As before, I consider a tabular presentation of the results to be more conducive to reading.

p. 8, l.175: It should be stated in which group the adverse effects occurred and how they were handled. It should be stated whether the effects were caused by the NFB or by the medication. This is not yet clearly formulated here.

p. 8, l. 183-186: Why is this mentioned?

p. 9, l. 189: the number of sessions should also be mentioned here, as this can have an important influence on effectiveness according to some previous studies.

p. 9, l. 190: reference is made here to results that are not so clearly emphasized in the results section.

p. 9, l.219: I would rather see this part in the introduction than in the benefit of deriving alternative explanations for the results of the study. The strengths and weaknesses of the study should also be weighed up and prospects for future studies given. These aspects are currently still missing or could be expanded.

p. 10, l. 233: In this paragraph, a clear distinction should be made between the conclusion and recommendations for future research.

A separate description of 'Participants' and 'Procedure' as well as 'Neurofeedback' and 'ATX HC' would be desirable to clarify the methods section. Overall, I recommend restructuring the results and discussion sections and formulating them more clearly. I also recommend ensuring standardized citation styles and formatting.
